# Superior Patellar Dislocation Misdiagnosed as Patellar Tendon Rupture: The Value of Ultrasonography

**DOI:** 10.1155/2016/2037381

**Published:** 2016-12-22

**Authors:** Artit Boonrod, Sermsak Sumanont, Manusak Boonard, Arunnit Boonrod

**Affiliations:** ^1^Department of Orthopedics, Faculty of Medicine, Khon Kaen University, 123 Mittraphap Road, Khon Kaen 40002, Thailand; ^2^Department of Radiology, Faculty of Medicine, Khon Kaen University, 123 Mittraphap Road, Khon Kaen 40002, Thailand

## Abstract

Superior dislocation of the patella with intact patellar tendon is a rare condition. Most cases in literatures were diagnosed by clinical examination and plain radiography; however there are many cases that were misdiagnosed as patellar tendon rupture. In this case, we demonstrate the use of ultrasound for diagnosis of superior dislocation of the patella in the emergency department. We also include a literature review of similar cases and discuss the advantages of different types of imaging for diagnosis in this condition.

## 1. Introduction

Superior dislocation of the patella with intact patellar tendon is a rare condition [1]. The age group of the patient is between 45 and 80 years. Most of them had preexisting degenerative joint [1–23]. Most cases in literature review were usually diagnosed by clinical examination and plain radiography. The primary superior dislocation of the patella is easily treated with simple closed reduction under adequate anesthesia. However, there are many cases in which that treatment was delayed due to misdiagnose as patellar tendon rupture [3, 4, 10, 17]. In this report, we describe and provide discussion on the ultrasound used, as a helpful additional imaging, for diagnosis of superior dislocation of the patella in the emergency department.

## 2. Case Presentation

**Figure 1:**
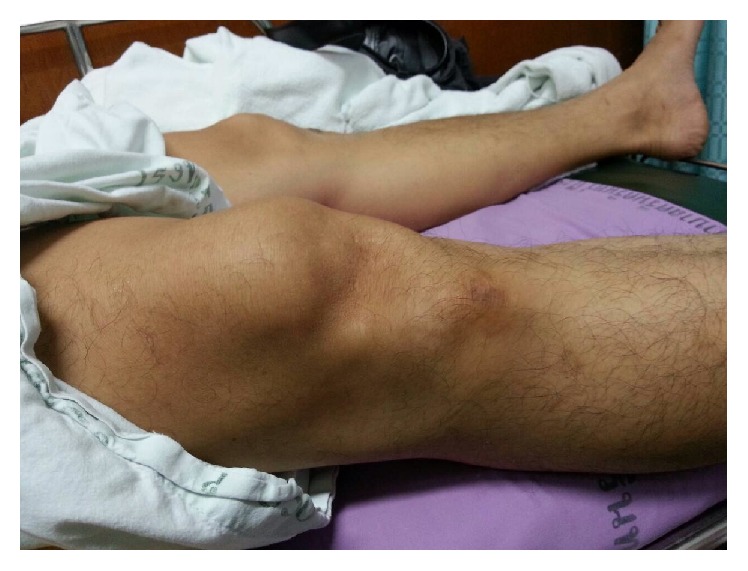
Clinical presentation with high-riding patella, anterior tilt of superior part of the patella, and skin dimple inferior to patella.

**Figure 2:**
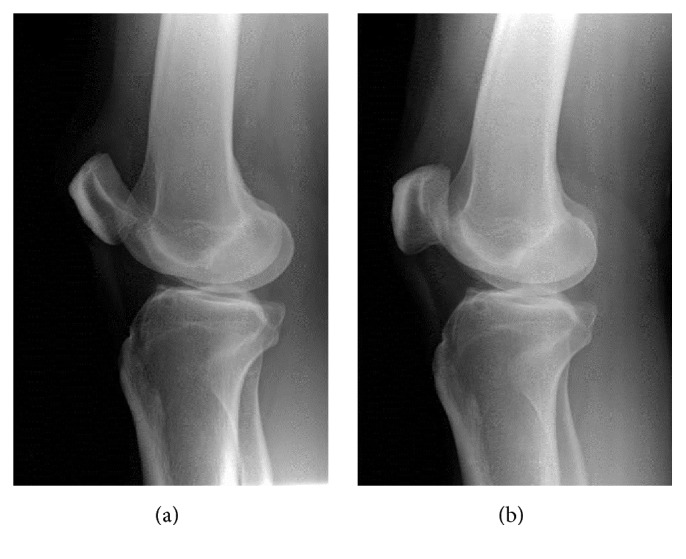
Pre- and postreduction lateral radiographs show superior dislocation of the patella. (a) showed high-riding patella with inferior patellar osteophyte locked to osteophyte at the superior aspect of femoral condyle. (b) showed normal position of patella.

**Figure 3:**
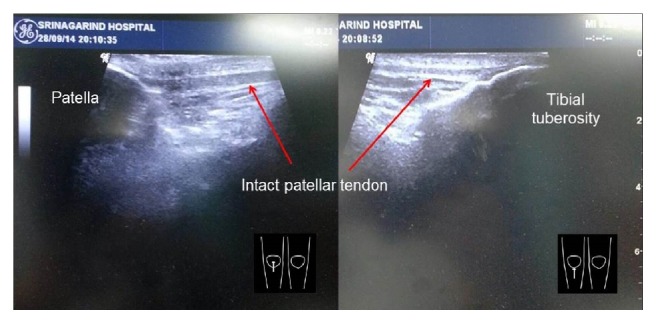
Under high frequency transducer (8 MHz linear transducer), the longitudinal scan showed multiple, parallel echogenic lines of the patellar tendon between the lower pole of the patella and the tibial tuberosity (arrowed). This ultrasound finding confirmed intact patellar tendon.

A 50-year-old man presented with right knee locked in hyperextension after falling with the knee extended. He had a severe knee pain and was unable to bend his knee. Initially, the patient was diagnosed as ruptured patellar tendon and was managed by immobilization with a long leg splint at a community hospital. The patient was referred to our hospital 5 hours after the injury. He had persistent anterior knee pain, inability to bend his knee, high-riding patella, anterior tilt of superior part of the patella, and skin dimple inferior to patella (Figure 1). The lateral radiograph of the right knee showed high-riding patella with inferior patellar osteophyte locked to osteophyte at the superior aspect of femoral condyle (Figures 2(a) and 2(b)). The physical examination and lateral radiographs of the knee represented superior patellar dislocation, but the patellar tendon integrity cannot be confirmed. Subsequently, the bedside ultrasonography, performed by an orthopedic surgeon, is used to evaluate the extensor mechanism of the knee. The patient was in supine position with his knee fixed in extension. Under high frequency transducer (GE healthcare, LOGIC Book, 8 MHz linear transducer), longitudinal ultrasound was performed through the entire length and width of the patellar tendon. Transverse scan was subsequently performed to complete the evaluation of patellar tendon in two perpendicular planes. The multiple, parallel echogenic lines of the patellar tendon were demonstrated between the lower pole of the patella and the tibial tuberosity (Figure 3). This ultrasound finding confirmed that the patellar tendon was intact. The dynamic study was not performed because the patient's knee was fixed in extension. The images were reviewed by a senior orthopedic surgeon and a radiologist, and the diagnosis of patellar dislocation with intact patellar tendon was agreed upon. After intravenous sedation, closed reduction was performed by using thumb and index finger to elevate the patella and gently move the patella into superior and lateral directions. The patella was easily relocated. The patient was able to achieve full active range of motion immediately after reduction. A follow-up ultrasound evaluation and lateral radiograph of the knee confirmed the anatomic reduction without any complication. Compression dressing was applied and partial weight walking with axillary crutch was advocated for 2 weeks. At 18 months' follow-up he had no pain or recurrent dislocation.

## 3. Discussion

Superior dislocation of the patella with intact patellar tendon is a rare condition, only 20 of which have been reported in English medical literature [1]. The first case was reported by Watson-Jones in 1956 [22]. Most patients with this condition had preexisting degenerative joint [1–23]. Osteophytes at the inferior aspect of patella and at the anterior aspect of medial condyle of femur are the identified cause of the locked knee in extension [7, 8, 12, 18, 19, 23]. The usual mechanism of injury is forceful contraction of quadriceps tendon with or without a posteriorly directed force [9].

However to make the correct diagnosis on the first visit might be difficult because most physicians are more familiar with lateral dislocation of the patella and rupture of the patellar tendon [1]. Many of the cases in previous studies received delayed treatment due to being misdiagnosed as patellar tendon rupture [3, 4, 10, 17]. Diagnosis should be differentiated from patellar tendon rupture because it is a surgical condition.

In most cases in literature, this condition is diagnosed by clinical examination and plain radiography. The presentations are anterior knee pain, locked knee in hyperextension, inability to bend knee, high-riding patella, anterior tilt of superior part of the patella, and skin dimple just inferior to the patella. Lateral radiograph shows superior dislocation of the patella and osteophytes at the inferior aspect of patella and at the anterior aspect of medial condyle of femur [4]. However, when the diagnosis is uncertain, additional imaging, such as ultrasound [4, 10], 3D-computed tomography [1], or magnetic resonance imaging [4, 9, 13], may be helpful (Table 1).

High-resolution ultrasonography has been recognized as an effective method of examining the extensor mechanism of the knee in both acute and chronic injuries. A key advantage of ultrasound over MRI is the ability to image tendons and bones dynamically and being useful for evaluating the reduction immediately in emergency department [4, 18]. Furthermore, in some cases, imaging is needed to look for any associated intra-articular damage such as osteophytes, osteochondral injury, or ligament tears. In this situation MRI provides a means of imaging of the intra-articular structures [4, 9, 13]. However, it is not recommended in the evaluation of most suspected extensor mechanism injuries because it is costly and usually unavailable in the emergency room. CT scan is useless imaging for diagnosis, because it is only shows the locked osteophytes [1], which can usually be clearly demonstrated by plain film.

In conclusion, although superior dislocation of the patella is a rare and non-life-threatening condition which may be unrecognized, a careful examination and investigation can lead to accurate diagnosis and appropriate management. At initial presentation, this condition may be confused with the rupture of the patellar tendon. Therefore, if physicians cannot exclude the rupture of the patellar tendon by clinical examination and plain radiograph, additional imaging studies such as ultrasound have an important role for correct diagnosis and confirmation of successful reduction immediately in the emergency department.

## Figures and Tables

**Table 1:** Additional imaging modalities.

Modality	Benefit
Ultrasound	(i) Evaluating the integrity of the patellar tendon [4, 10, 18]. (ii) Evaluating the medial structure injury such as vastus medialis tear [4]. (iii) Follow-up immediately after closed reduction [4, 18].

3D-computed tomography	(i) Evaluating the osteophytes at the inferior aspect of the patella and at the superior aspect of femoral condyle [1].

Magnetic resonance imaging	(i) Evaluating the integrity of the patellar tendon [4, 9, 13]. (ii) Evaluating the osteophytes at the inferior aspect of the patella and at the superior aspect of femoral condyle [4, 9, 13]. (iii) Evaluating any associated injury such as vastus medialis tear [4]. (iv) Evaluating the cartilage and other intra-articular pathologies.

## References

[1] Gakhar H., Singhal A. (2013). Superior dislocation of the patella: case report and review of the literature. *Journal of Emergency Medicine*.

[2] Bartlett D. H., Gilula L. A., Murphy W. A. (1976). Superior dislocation of the patella fixed by interlocked osteophytes. A case report and review of the literature. *The Journal of Bone & Joint Surgery—American Volume*.

[3] Bassi R. S., Kumar B. A. (2003). Superior dislocation of the patella; a case report and review of the literature. *Emergency Medicine Journal*.

[4] Clift R. K., El-Alami W. (2012). Superior patellar dislocation: the value of clinical examination and radiological investigation. *BMJ Case Reports*.

[5] Cusco X., Seijas R., Ares O., Cugat J. R., Garcia-Balletbo M., Cugat R. (2009). Superior dislocation of the patella: a case report. *Journal of Orthopaedic Surgery and Research*.

[6] Friden T. (2009). A case of superior dislocation of the patella. *Acta Orthopaedica Scandinavica*.

[7] Hansen B., Beck C., Townsley R. (2003). Arthroscopic removal of a loose body osteophyte fragment after superior patellar dislocation with locked osteophytes. *Arthroscopy*.

[8] Harris N., Hay S., Bickerstaff D. (1995). Recurrent traumatic superior dislocation of the patella with interlocking osteophytes. *The Knee*.

[9] Iorwerth A., Thomas R., Shewring D. J. (2001). Confirmation of an intact patellar tendon in superior dislocation of the patella using magnetic resonance imaging. *Injury*.

[10] Joseph G., Devalia K., Kantam K., Shaath N. M. (2005). Superior dislocation of the patella. Case report and review of literature. *Acta Orthopaedica Belgica*.

[11] Lai R., Lau Y. K. (2007). Superior dislocation of the patella treated by closed reduction: a rare case report and review of the literature. *Hong Kong Journal of Emergency Medicine*.

[12] McWilliams T. G., Binns M. S. (2000). A locked knee in extension: a complication of a degenerate knee with patella alta. *Journal of Bone and Joint Surgery B*.

[13] Ofluoglu O., Yasmin D., Donthineni R., Yildiz M. (2006). Superior dislocation of the patella with early onset patellofemoral arthritis: a case report and literature review. *Knee Surgery, Sports Traumatology, Arthroscopy*.

[14] Rao J. P., Meese M. A. (1997). Irreducible superior dislocation of the patella requiring open reduction. *American Journal of Orthopedics*.

[15] Roth R. M., McCabe J. B. (1985). Nontraumatic superior dislocation of the patella. *Journal of Emergency Medicine*.

[16] Saleemi A. J., Hussain A., Iqbal M. J., Thuse M. G., George A. A. (2007). Superior dislocation of patella in a rugby player: an update on a extremely rare condition and review of literature. *Knee Surgery, Sports Traumatology, Arthroscopy*.

[17] Scott S. J., Molloy A., Harvey R. A. (2000). Superior dislocation of the patella—a rare but important differential diagnosis of acute knee pain—a case report and review of the literature. *Injury*.

[18] Siddiqui M. A., Tan M. H. (2011). Locked knee from superior dislocation of the patella-diagnosis and management of a rare injury. *Knee Surgery, Sports Traumatology, Arthroscopy*.

[19] Siegel M. G., Mac S. S. (1982). Superior dislocation of the patella with interlocking osteophytes. *Journal of Trauma*.

[20] Takai S., Yoshino N., Hirasawa Y. (1998). Arthroscopic treatment of voluntary superior dislocation of the patella. *Arthroscopy*.

[21] Teuscher D. D., Goletz T. H. (1992). Recurrent atraumatic superior dislocation of the patella: case report and review of the literature. *Arthroscopy*.

[22] Watson-Jones R. (1956). *Fractures and Joint Injuries*.

[23] Yip D. K., Wong J. W., Sun L. K., Wong N. M., Chan C. W., Lau P. Y. (2004). The management of superior dislocation of the patella with interlocking osteophytes—an update on a rare problem. *Journal of orthopaedic surgery*.

